# Dietary Conjugated Linoleic Acid and Hepatic Steatosis: Species-Specific Effects on Liver and Adipose Lipid Metabolism and Gene Expression

**DOI:** 10.1155/2012/932928

**Published:** 2011-08-22

**Authors:** Diwakar Vyas, Anil Kumar G. Kadegowda, Richard A. Erdman

**Affiliations:** ^1^Department of Animal and Avian Sciences, University of Maryland, College Park, MD 20742, USA; ^2^Department of Animal and Veterinary Sciences, Clemson University, Clemson, SC 29634, USA

## Abstract

*Objective*. To summarize the recent studies on effect of conjugated linoleic acid (CLA) on hepatic steatosis and hepatic and adipose lipid metabolism highlighting the potential regulatory mechanisms. *Methods*. Sixty-four published experiments were summarized in which *trans*-10, *cis*-12 CLA was fed either alone or in combination with other CLA isomers to mice, rats, hamsters, and humans were compared. *Summary and Conclusions*. Dietary *trans*-10, *cis*-12 CLA induces a severe hepatic steatosis in mice with a more muted response in other species. Regardless of species, when hepatic steatosis was present, a concurrent decrease in body adiposity was observed, suggesting that hepatic lipid accumulation is a result of uptake of mobilized fatty acids (FA) from adipose tissue and the liver's inability to sufficiently increase FA oxidation and export of synthesized triglycerides. The potential role of liver FA composition, insulin secretion and sensitivity, adipokine, and inflammatory responses are discussed as potential mechanisms behind CLA-induced hepatic steatosis.

## 1. Introduction

Obesity is a chronic metabolic nutritional disorder that has increased at an alarming rate in the last 20 years [1]. In the US, 68% of the adults (age ≥ 20 years) and 18% of children (2–19 years) are either obese or overweight as per the National Health and Nutrition Examination Survey [2, 3]. Incidence of obesity is associated with many health complications such as hypertension, hyperlipidemia, cardiovascular disease, type 2 diabetes [4], and a range of lipid abnormalities, the most common being nonalcoholic fatty liver disease (NAFLD) [4]. 

NAFLD is an important health concern due to its high prevalence (~20% of adult population) and its association with insulin resistance and metabolic syndrome [5]. It is characterized by hepatic lipid accumulation primarily in the form of triglycerides (TG) [6]. Some of the potential steps involved in the progression of NAFLD may involve increased uptake of circulating fatty acids (FA) [7], increased hepatic denovo lipogenesis (DNL) [8], reduced rate of FA oxidation [9], or reduced FA secretion [10, 11]. When NAFLD is associated with inflammation and fibrosis, it is termed as nonalcoholic steatohepatitis (NASH), a serious condition that could lead to liver cirrhosis, hepatic carcinoma, and liver failure [12]. The pathogenesis of NAFLD can be explained by “two hit” hypothesis suggesting steatosis as the “first hit” which increases the vulnerability of liver to various second hits like oxidative stress and inflammation leading to NASH [13]. 

Although no specific guidelines exist for treatment of NAFLD, recommendations are aimed at reducing body weight due to its strong association with obesity and metabolic syndrome [4]. In this regard, bioactive lipids/FA as functional food may be important in modulating metabolism and body weight. A specific group of polyunsaturated FA collectively known as conjugated linoleic acid (CLA) have been suggested to have an effect on regulating energy metabolism [14] and is being used commercially as a weight-loss supplement. CLA were recently granted “Generally Recognized As Safe”, status in the United States (GRN no. 232; http://www.cfsan.fda.gov/) for use as a dietary supplement. However, CLA effects are varied depending on the type of CLA isomer, the animal's physiological condition, and the tissue type examined. In this paper, we summarize the recent studies on effect of CLA on hepatic lipid metabolism highlighting the potential regulatory mechanisms.

## 2. Conjugated Linoleic Acid

Conjugated linoleic acid refers to a group of dienoic derivatives of linoleic acid with conjugated double bonds arranged in different combinations of *cis* and *trans* configuration [15]. Currently, 16 naturally occurring CLA isomers have been identified with different positional (7/9, 8/10, 9/11, 10/12, and 11/13) and geometric (*cis*/*cis*, *trans*/*trans*, *cis*/*trans*, and *trans*/*cis*) combinations [16, 17]. 

Sources of CLA include those naturally present in dairy products and meat from ruminant animals or those contained in industrially hydrogenated vegetable oils and other synthetic products [14]. The CLA originating from the ruminant products predominantly consist of *cis*-9, *trans*-11 CLA (>80%), with a small amounts of *trans*-10, *cis*-12 CLA and other isomers [18]. The industrially synthesized CLA and other commercial products intended for human consumption typically consists of equal amounts of *cis*-9, *trans*-11 CLA and *trans*-10, *cis*-12 CLA and other isomers [19]. Of all the CLA isomers, *cis*-9, *trans*-11 CLA and *trans*-10, *cis*-12 CLA have been the most widely studied due to their biologically active properties [15].

## 3. Physiological Effects of CLA

A great deal of current interest in CLA is due to their bioactive properties including anticarcinogenic [19], antiatherogenic [20], immunity enhancing [21], and effects on body composition [22]. Each CLA isomer has unique bioactive properties, and hence, the biological effect from a mixture of dietary CLA isomers, as is the case in most of the studies, would be the combined effect of their distinct isomers [15]. For example, *cis*-9, *trans*-11 CLA and *trans*-10, *cis*-12 CLA have additive effects on cancer [23] and immune cell functions [24] but are antagonistic on insulin sensitivity. While *cis*-9, *trans*-11 CLA improves insulin sensitivity, *trans*-10, *cis*-12 CLA causes insulin resistance. Also, *trans*-10, *cis*-12 CLA is solely responsible for changes in body composition and reducing adipose mass [25].

### 3.1. Body Weight and Lean Mass

 CLA reduces body weight and body fat mass and increases lean mass in different species [22]. However, the response appears to vary depending on species, physiological stage, and fat depot [22, 26]. Table 1 provides a summary of studies reviewed across species with respect to body weight and adiposity, where the number of experiments showing significant (*P* > 0.05) increases, decreases, or no change and the mean response to dietary *trans*-10, *cis*-12 CLA within those categories are reported. The range of *trans*-10, *cis*-12 CLA addition in these studies varied between 0.1 and 1 percent of the diet.

*Trans*-10, *cis*-12 CLA reduces body fat to a maximum extent in mice (60% to 80%) [27, 28]. However, modest and inconsistent effects are seen in rats [49, 82], hamsters (9% to 58%) [54, 55], and pigs (6% to 25%) [83]. Similarly, variable responsiveness to CLA was observed for epididymal, perirenal, and subcutaneous body fat depots [55]. Inconsistent responses to *trans*-10, *cis*-12 CLA have been reported in clinical trials with humans [84]. Some have shown significant effects on body composition [63, 85], while others have not [64, 65]. The differences in the responses are attributed to differences in the dose levels, age, and rate of adipose tissue TG turnover [14, 66, 84]. The response to CLA isomers also depends on the physiological state of the animal which is probably due to differences in the preferential uptake of CLA by different tissues. For example, *trans*-10, *cis*-12 CLA is preferentially taken up by the mammary tissue during lactation leading to substantial (~45%) decrease in milk lipid synthesis [29].

### 3.2. Effects of CLA on Hepatic Lipid Metabolism

**Figure 1:**
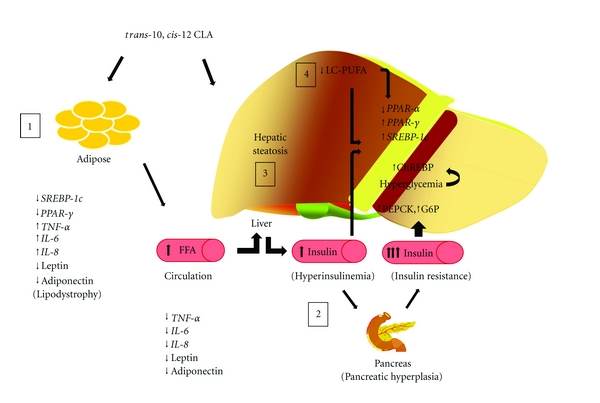
Current concepts in the pathways of *trans*-10*, cis*-12 CLA-induced hepatic steatosis. (1) Adipose tissue lipodystrophy caused by increased proinflammatory cytokines and reduced adipokines leading to higher circulatory levels of free FA (FFA). (2) Hyperinsulinemia induced by systemic insulin resistance. (3) Alterations in hepatic lipid metabolism leading to hepatic steatosis. (4) Alterations in hepatic FA composition. *SREBP-1c, Sterol regulatory element-binding protein-1c; PPAR-*γ*, peroxisome proliferator activated receptor-*γ*; TNF-*α*, tumor necrosis factor-*α*; IL-6, interleukin-6; IL-8, interleukin-8; PEPCK, phosphoenol pyruvate carboxykinase; G6P, glucose 6-phosphatase; ChREBP, carbohydrate response element-binding protein; PPAR-*α*, peroxisome proliferator-activated receptor-*α*;* LC-PUFA, long chain polyunsaturated FA.

Liver plays an important role in energy homeostasis, as it converts excessive dietary glucose into FA which is exported as TG. Liver is an important target for CLA effects irrespective of the physiological condition. Of the different CLA isomers, *trans*-10, *cis*-12 CLA causes increased lipid accumulation leading to hepatic steatosis [30–32, 86]. However, the intensity of lipid accumulation varies depending on the level of CLA in the diet, duration of feeding, physiological condition, and animal species (Table 1). The factors leading to hepatic lipid accumulation are multifactorial involving increased FA influx, increased FA synthesis, and altered FA oxidation and TG secretion insufficient to prevent lipid accumulation (Figure 1) [33]. These mechanisms are probably not mutually exclusive and could act in a coordinated manner to hasten the development and progression of fatty liver [87]. 

#### 3.2.1. Hepatic FA Synthesis

 Under normal conditions, *de novo *lipogenesis contributes minimally to the lipid pool in the liver [88]. However, the lipid synthesis increases to as much as 26% during steatotic conditions [89]. The increase in hepatic lipid content due to CLA, specifically *trans*-10, *cis*-12 CLA, is commonly associated with increased hepatic lipogenesis [30]. In mice, CLA has been repeatedly shown to increase the expression of *sterol regulatory element-binding protein-1c (SREBP-1c)*, key transcriptional regulator in hepatic lipogenesis and its downstream genes *acetyl CoA carboxylase (ACC), fatty acid synthase (FASN), and stearoyl CoA desaturase-1 (SCD1)* [30, 34, 35] (Table 2). However, in rats and hamsters, the responses are equivocal. The increase in *SREBP-1c* expression in mice is attributed to hyperinsulinemia (Figure 1) [30]. The decreased expression of lipogenic* (ACC1, ACC2, FASN,* and *SCD1*) genes in the absence of insulin in mice fed *trans*-10, *cis*-12 CLA further supports this argument [33]. In addition to *SREBP-1c*, insulin induces the expression of *peroxisome proliferator-activated receptor-*γ* (PPAR-*γ*)* [90], which is in low abundance under normal conditions [91]. *PPAR-*γ** expression is increased in steatotic liver (Figure 1) [30, 92], while its ablation ameliorates the condition in mice [93]. Insulin resistance in response to *trans*-10, *cis*-12 CLA could upregulate genes of glucogenic pathway (e.g., *PEPCK, G6P*) leading to hyperglycemia (Figure 1) [94]. In turn, elevated blood glucose concentrations could upregulate hepatic lipogenesis through *carbohydrate response element binding protein* (*ChREBP)*, a transcriptional regulator modulated by glucose (Figure 1). The targeted deletion of *ChREBP* in the liver improves the steatotic conditions in *ob/ob* mice [94]. However, the role of *ChREBP* in CLA-induced hepatic steatosis is not known. Although hyperinsulinemia triggers the hepatic lipogenesis, CLA-induced hepatic steatosis in the absence of insulin suggests the involvement of other regulatory mechanisms affecting hepatic lipid accumulation [33].

#### 3.2.2. Hepatic FA Uptake and TG Secretion

 In mouse experiments, dietary *trans*-10, *cis*-12 CLA was associated with upregulation of genes associated with FA uptake and TG secretion *(FAT/CD36*; Table 2). During hepatic steatosis about 59% of hepatic TG is derived from free FA released from the adipose tissue and 15% is derived from dietary fat [89]. FA transporters, (*FATP5, FAT/CD36, FABP-1, FABP-4,* and *FABP-5*) regulate the FA uptake by hepatocytes. While the overexpression of these proteins promotes steatosis, functional deletion ameliorates the condition [98–100]. As CLA are natural ligands and activators of *PPAR*-**γ** [101] the upregulation of *FAT/CD36* by *trans*-10, *cis*-12 CLA [32, 33, 102] could be through *PPAR*-**γ** leading to increased hepatic FA uptake. In addition to *FAT/CD36*, we have observed modest increases in the expression of *FABP-1* (1.39 fold) and *FABP-2* (1.7 fold) in liver of lactating mice fed *trans*-10, *cis*-12 CLA (Kadegowda, A. K. G., Erdman, R. A., and Loor, J. J., unpublished results). 

Besides enhanced FA uptake and lipogenesis, alteration in very low-density lipoprotein (VLDL) secretion rates could also result in liver fat accumulation [103]. The VLDL production and secretion is increased in response to elevated lipid concentrations. However, impaired or insufficient fat export via VLDL predisposes animal to hepatic steatosis [10]. *Trans*-10, *cis*-12 CLA reduced TG secretion leading to higher lipid accumulation in HepG2 cells due to reduced apolipoprotein B synthesis [104]. Conversely, lipoprotein clearance was not affected in mice fed CLA [31, 102]. The TG export was increased with higher rate of VLDL secretion; however, it was insufficient to eliminate increased FA flux entering the liver leading to hepatic steatosis [31]. 

#### 3.2.3. Hepatic FA Oxidation

Hepatic FA oxidation encompasses *β*-oxidation in mitochondria and peroxisomes and *ω*-oxidation in the microsomes [105]. The FA < C8 to C20 are catabolized through the mitochondrial *β*-oxidation pathway, while FA > C20 are initially catabolized in the peroxisomes to shorter FA which are then shuttled to mitochondria for further oxidation [32]. Previous studies have reported variable responses in hepatic FA oxidation with *trans-*10, *cis-*12 CLA. Most of the studies have shown increased FA oxidation [27, 34, 36, 56, 106], while some have reported reduced [32] or unaltered FA oxidation [22] with CLA.

*Carnitine palmitoyltransferase-1 (CPT1)* is the rate limiting enzyme for mitochondrial *β*-oxidation pathway, as it regulates the transport of fatty acyl CoA into mitochondria. When measured in mice, *CPT1* gene expression was consistently increased by CLA (Table 2) which might be mediated through transcriptional regulator PPAR-*α* as it regulates the key enzymes (e.g.,* CPT1, CPT2, *and* ACO*) involved in hepatic FA oxidation [50]. 

Despite increased FA oxidation hepatic steatosis was consistently observed in mice (Tables 1 and 2). Since studies showing increased FA oxidation were also associated with increased hepatic lipogenesis, it is possible that that the rates of hepatic lipogenesis far exceed the rates of FA oxidation resulting in increased lipid accumulation. Along with increased lipogenesis the level of malonyl CoA, a product of *ACC*, was also increased that allosterically inhibits *CPT1* enzyme activity [36]. Thus, despite higher expression of FA oxidation genes, it is possible that FA combustion might be depressed *in vivo* leading to steatosis. 

Some studies have shown CLA induced downregulation of genes related to mitochondrial *β*-oxidation (*CPT1*), and *ω* oxidation (*cyt P450* and *FMO3*) [32]. We have also observed decreased expression of *CPT1*, *ACOX1,* and *FMO3* without any changes in hepatic lipogenic genes of lactating mice fed* trans*-10, *cis*-12 CLA (Kadegowda, A. K. G., Erdman, R. A., and Loor, J. J., unpublished results). The variable responses among different studies can be attributed to the level and type of fat used in the experimental diet along with the physiological conditions of animal used in the experiment. 

#### 3.2.4. Effect of CLA on Hepatic FA Composition

*Trans*-10, *cis*-12 CLA-induced hepatic steatosis is characterized by changes in hepatic FA composition [29, 37, 107–111] similar to those induced during NAFLD [112]. The hepatic FA composition in steatotic liver determines the extent of susceptibility of liver injury [113]. The steatotic liver FA profile is characterized by substantial reductions in long chain polyunsaturated FA (LC-PUFA) concentrations; specifically that of arachidonic acid (C20:4n-6). While linoleic (18:2n-6) and *α*-linolenic acid (18:3n-3) are unaltered, the concentrations of eicosapentaenoic acid (EPA, C20:5n-3) and docosahexaenoic acid (DHA, C22:6n-3) are decreased. The desaturation and elongation of linoleic and *α*-linolenic acid by desaturases (Δ^5^-desaturase, Δ^6^-desaturase) and elongases (ELOVL-2, ELOVL-3) are involved in synthesis of LC-PUFA. *Trans*-10, *cis*-12 CLA inhibits both Δ^5^- and Δ^6^-desaturase in HepG2 cells [114]. A recent tracer study with [U-^13^C] linoleic acid showed significant reduction in n-6 PUFA synthesis by inhibition of elongation and desaturation in the liver homogenates of neonatal pigs [115]. A decrease in arachidonic acid synthesis would alter eicosanoid metabolism and potentially reduce the synthesis of prostaglandin E2 (PGE_2_) [116] which is known to have protective effects on liver [117].

Typical NAFLD is also characterized by increased n-6 : n-3 LC-PUFA ratio which favors lipid synthesis over lipid oxidation and secretion leading to hepatic lipid accumulation [118]. *Trans*-10, *cis*-12 CLA reduces the n-3 PUFA in liver [38, 109] in addition to arachidonic acid. The n-3 PUFA downregulate *SREBP-1c* and upregulate *PPAR-*α*,* which regulates lipid oxidation *(CPT1, ACOX1)* and secretion *(ApoB100)*. A decrease in hepatic n-3 PUFA would not only reduce lipid oxidation but increase lipogenesis leading to hepatic steatosis [118]. Although the *trans-*10, *cis*-12 CLA-induced responses in FA oxidation are variable in mice, consistently increased lipogenesis (Table 2) suggests a potential role for n-3 PUFA. On the contrary, CLA feeding increased n-3 PUFA content and decreased n-6 PUFA in the rats [119, 120] which could probably explain the differences in CLA effects between the two species. Although the exact mechanism of CLA action has not been elucidated, Banni et al. [121] has suggested that the metabolites of CLA, conjugated dienes (CD)18:3, CD20:3, CD20:4, could compete with other PUFA at the level of formation and metabolism in liver and affect LC-PUFA synthesis. 

### 3.3. CLA and SCD in Hepatic Lipid Metabolism

In the adipose, there are some similarities between the effects of *trans*-10, *cis*-12 CLA and the inhibition of *SCD1*. For example, reduced adiposity is observed with both dietary *trans*-10, *cis*-12 CLA and *SCD1* inhibition and one could speculate that the effects of *trans*-10, *cis*-12 CLA are mediated through *SCD1* as *trans*-10, *cis*-12 CLA decreases *SCD1* in adipose [122]. However, a study with *SCD1^−/−^* mice showed that the antiobesity effects of *trans*-10, *cis*-12 CLA were independent of *SCD1* gene expression and enzyme activity [123]. 

 Unlike adipose, the effects of *trans*-10, *cis*-12 CLA are varied in liver (Table 2). While *trans*-10, *cis*-12 CLA decreased hepatic *SCD* activity *in vitro* [124], *in vivo* studies report increased hepatic *SCD1* gene expression [32, 95]. In contrast to *trans*-10, *cis*-12 CLA effects in mice, *SCD1^−/−^* mice showed increased insulin sensitivity, reduced hepatic lipogenic genes, upregulated lipid oxidizing genes, increased hepatic saturated FA and unchanged hepatic n-3 and n-6 PUFA [125]. *SCD1^−/−^* mice fed *trans*-10, *cis*-12 CLA showed reduced hepatic accumulation compared to wild type [123] confirming that reduced *SCD1* expression decreases hepatic lipid accumulation [126]. Liver specific *SCD1* knock out decreased expression of *SREBP1* and *ChREBP* and their target genes there by reducing hepatic lipogenesis [127]. In contrast, short-term inhibition of tissue specific hepatic *SCD* increased hepatic TG content and enhanced insulin signaling, [128] but the long-term inhibition decreased hepatic steatosis [129]. The differences in responses observed in liver specific knockout versus complete *SCD* knockout mice suggests that hepatic lipid metabolism is being affected by lipid metabolism in nonhepatic tissues [130].

 As *trans*-10, *cis*-12 CLA effects in mice are mostly associated with insulin resistance; increased hepatic *SCD1* expression is probably due to increased *SREBP-1c* expression. Hepatic steatosis due to *trans*-10, *cis*-12 CLA is also seen in the absence of insulin and is associated with reduced expression of *SCD1* and other lipogenic genes [33]. These results indicate that the disturbances in hepatic lipid metabolism caused by dietary *trans*-10, *cis*-12 CLA are mediated by multiple mechanisms [131] rather than through changes in *SCD1* alone. 

### 3.4. Role of Adipose during CLA-Induced Hepatic Steatosis

The effect of CLA on adipose lipid metabolism is well documented [14]. Of all the CLA isomers, *trans*-10, *cis*-12 CLA is the most potent to induce changes in adipose [25]. The changes may be caused by reduced lipid content, size, and number of adipocytes. *Trans*-10, *cis*-12 CLA reduces lipogenesis by decreasing expression of *SREBP-1c* and *PPAR-*γ** and their downstream genes, *ACC*, *FASN*, and *SCD1*, reduces glucose and FA uptake by reducing *GLUT4* and *LPL* expression, increases FA oxidation by increasing *CPT1* and *UCP2* expression and reduces adipocyte proliferation and differentiation by reducing *PPAR-*γ**and its downstream genes [131]. Furthermore, CLA affects various adipocyte secreted-adipokines (e.g., leptin, adiponectin, and resistin) and cytokines (e.g., *TNF*α** and *IL6*), which are involved in wide range of physiological activities [14]. *Trans*-10, *cis*-12 CLA increases the mRNA expression of cytokines, *TNF*α** and *IL6* in adipose tissue [131]. However, the circulating cytokine levels are reduced in response to *trans-*10*, cis-*12 CLA (Table 2). The increased cytokine expression in adipose tissue is known to reduce the activity of *PPAR-*γ** [132], and thereby affect its downstream cellular functions. Also, *TNF*α** and *IL6* inhibit the activation of insulin receptor substrate-1 (*IRS-1*) through induction of suppressors of cytokine signaling (SOCS3) disrupting insulin action [133]. The expressions of *TNF*α** and adiponectin, an adipokine associated with insulin sensitivity, are inversely related [134]. The adipose tissue depletion would reduce the level of adiponectin and when coupled with increased *TNF*α** would lead to severe insulin resistance. The subsequent pancreatic *β* cell hyperplasia, as a compensatory mechanism to insulin resistance, leads to hyperinsulinemia which promotes lipid accretion in the liver leading to hepatic steatosis [39]. 

In mice, *trans*-10, *cis*-12 CLA causes severe lipodystrophy reducing the levels of leptin and adiponectin (Table 2), which leads to hepatic steatosis (Table 1, Figure 1). Re-establishing the levels of leptin or adiponectin either through external supplementation (in case of leptin) or induction using rosiglitazone (ROSI) (in case of adiponectin) attenuated hepatic steatotic condition and normalized the insulin levels in CLA-fed mice [135, 136]. Similar results are seen in studies where prevention of lipodystrophy prevented lipid accumulation in the liver [135]. Serum insulin levels are directly correlated with liver TG, while serum adiponectin levels are inversely related [35]. Adipokines could improve the condition of the liver by lowering the insulin levels. However, hepatic steatosis is seen in mice even at low insulin levels [33], suggesting that different mechanisms could regulate the induction of hepatic steatosis depending on the animal's physiological condition. The intensity of hepatic steatosis could be directly related to the relative amounts of adipose tissue. CLA-induced hyperinsulinemia and hepatic steatosis are observed only if there are corresponding decreases in the adipose tissue mass [40, 135]. Stout et al. [137], reported increases in diacylglycerol (DAG) concentration and membrane associated *protein kinase C (PKC)* during *trans*-10, *cis*-12 CLA-induced hepatic steatosis. Increased *PKC* would affect insulin signaling leading to insulin resistance, hyperinsulinemia, and hyperglycemia [137]. 

### 3.5. CLA and Inflammatory Responses

In addition to its effects on lipid metabolism, *trans*-10, *cis*-12 CLA also induces an inflammatory response in adipose tissue [131, 138]. *Trans*-10, *cis*-12 CLA activates integrated stress response leading to activation of NF-kB pathway, induction of inflammatory cytokines, *TNF*α**, *IL6*, and *IL8* [41, 138, 139], and macrophage infiltration [35]. However, the level of circulating cytokines, *TNF*α** and *IL6*, were decreased in response to *trans*-10, *cis*-12 CLA [42, 43]. In contrast to the adipose, the effects of CLA on hepatic inflammatory responses are not well defined. *Trans*-10, *cis*-12 CLA did not affect expression markers of macrophage infiltration in mice liver such as *TNF*α** or *F4/80* and *CD68* during hepatic steatosis [35]. However, *trans*-10, *cis*-12 CLA increased expression of markers of hepatic inflammation in hamsters without inducing hepatic steatosis [57]. The authors in [57] attributed this to an increased capacity of the liver for higher FA oxidation leading to inflammation and oxidant stress defense pathway in the hamsters.

## 4. Prevention or Amelioration of CLA-Induced Hepatic Steatosis

Several studies have examined either the prevention or amelioration of *trans*-10, *cis*-12 CLA-induced hepatic steatosis (Table 3) by normalizing serum adipokine levels, altering hepatic PUFA composition or both. External supplementation of recombinant murine leptin ameliorate CLA-induced hepatic steatosis and hyperinsulinemia by decreasing hepatic lipogenesis and increasing insulin sensitivity respectively [40, 136]. Serum adiponectin levels were not restored (and remained low) even after leptin supplementation, prompting the authors in [136] to claim that leptin alone could ameliorate CLA induced steatosis. Conversely, *trans*-10, *cis*-12 CLA-caused hyperinsulinemia associated with lipid steatosis in *Ob/Ob* mouse which lack functional leptin [143] suggests the involvement of other factors. Increasing adiponectin levels by supplementation of ROSI attenuates liver fat accumulation in *Ob/Ob* mouse [49]. ROSI prevented lipodystrophy, decreased hepatic lipogenesis and subsequently liver TG content [35]. The insulin sensitizing action of leptin and adiponectin normalizes insulin levels which further helps in preventing CLA-induced steatosis [40, 141]. 

Dietary FA or oil supplements with higher n-3 and n-6 PUFA are able to ameliorate liver steatosis when supplemented along with CLA. Supplementing arachidonic acid [140] or its precursor *γ*-linolenic acid (18:3n-6) [44] decreased induction of hepatic steatosis and increased liver PGE_2_ levels. Hepatic steatosis is characterized by significant reduction in the levels of arachidonic acid in liver. Arachidonic acid supplementation would not only normalize the level of respective FA but would also increase the levels of hepatic PGE_2_ [44, 140]. Both arachidonic acid and PGE_2_ would further reduce hepatic lipogenesis by decreasing *FASN* and *S14* gene expression [140, 144] thereby preventing hepatic steatosis. 

The importance of n-3 PUFA concentrations on hepatic lipid metabolism was explained in the earlier section. *Trans*-10, *cis*-12 CLA decreases liver n-3 PUFA concentrations which affect hepatic lipid metabolism. Dietary supplements enriched in n-3 PUFA along with CLA diet increased the content of n-3 and n-6 PUFA in liver [38]. Fish oil, a source of PUFA has been shown to ameliorate CLA-induced steatosis by increasing leptin and adiponectin levels and decreasing plasma insulin [27]. Pinolenic oil, a source of Pinolenic acid was able to stabilize insulin levels when fed with CLA [141]. Similarly, flaxseed oil, a source of *α*-linolenic acid was able to increase n-3 and n-6 PUFA in liver. Supplementing EPA and DHA prevents lipid accumulation when fed with *trans*-10, *cis*-12 CLA [45, 142]. This effect was independent of their effects on stabilizing insulin sensitivity. Both EPA and DHA have modest effects in restoring plasma leptin levels, while DHA alone can restore plasma adiponectin levels to some extent [142]. The effects of DHA in preventing hepatic steatosis were mediated through decreasing hepatic lipogenesis [45]. 

## 5. Role of *Cis-*9, *Trans-*11 CLA in Hepatic Metabolism

Of the 16 naturally occurring CLA isomers, *trans*-10, *cis*-12 CLA and *cis*-9, *trans*-11 CLA have been the most extensively studied with respect to their bioactive properties. Most of the animal studies have used a CLA mixture having *trans*-10, *cis*-12 CLA and *cis*-9, *trans*-11 CLA in 1 : 1 ratio to study the effect of CLA on liver metabolism. Studies using purified CLA isomer have delineated the differences between the two isomers. While *trans*-10, *cis*-12 CLA leads to decreased adipose tissue leading to insulin resistance, hyperinsulinemia, and hepatic steatosis,* cis*-9, *trans*-11 CLA shows only modest effects in mice [30–32, 86] and hamsters [56, 58]. Similarly, the effects of CLA on *SCD1* gene and protein expression are isomer specific [145]. Contrary to *trans*-10, *cis*-12 CLA, *cis*-9, *trans*-11 CLA has no effect on *SCD1* gene expression either *in vitro* [124] or *in vivo* [95]. 

 A few studies have reported beneficial effects of *cis*-9, *trans*-11 CLA. For example, *cis*-9, *trans*-11 CLA did not alter liver lipid content but reduced 18:1n-9 and 18:1n-7 and increased 18:2n-6 in TG in contrast to *trans*-10, *cis*-12 CLA [108]. In addition, *cis*-9, *trans*-11 CLA promotes insulin sensitivity [42, 43] by reducing adipose inflammation [41, 132]. Furthermore, it enhances hepatic mitochondrial function and protects against oxidative stress by increasing activities of mitochondrial antioxidant enzymes [146]. The anti-inflammatory role of *cis*-9, *trans*-11 CLA is related to the induction of anti-inflammatory heat shock protein (HSP) 70 kDa and decreased expression of proinflammatory macrophage migration inhibitory factor [147].

## 6. Conclusions

Hepatic steatosis induced by *trans*-10, *cis*-12 CLA is associated with lipodystrophy in addition to insulin resistance, hyperinsulinemia, and hyperglycemia in mice (Figure 1). These effects are largely attributed to decreased adipokine (leptin and adiponectin) secretion. Dietary interventions preventing lipodystrophy or normalizing leptin and adiponectin levels prevents or ameliorates hepatic steatosis in mice, suggesting that adipose tissue responsiveness to *trans*-10, *cis*-12 CLA could be the main contributing factor. The moderate responsiveness of adipose tissue to *trans*-10, *cis*-12 CLA observed in hamsters and rats results in lower (or absence of) hepatic TG accumulation when compared with mice (Table 1) explains species specific responses. 

Hepatic steatosis, due to increased lipid accumulation, is multifactorial and is largely attributed to increased rates of lipid synthesis along with lipid uptake, and it far exceeds the rates of FA oxidation and VLDL secretion. In addition, *trans*-10, *cis*-12 CLA-induced hepatic steatosis is characterized by reduction of n-6 PUFA (especially C20:4n-6) and n-3 PUFA (Figure 1). Changes in hepatic FA composition could play an important role in progression of hepatic steatosis, as normalizing the levels of n-6 PUFA or n-3 PUFA by dietary supplementation prevents or ameliorates hepatic lipid accumulation. Further studies are needed to understand the molecular mechanisms and the interrelationship between *trans*-10, *cis*-12 CLA-induced hepatic steatosis and altered hepatic PUFA content. We are still lacking mechanistic details showing relationship between adipokine levels, insulin resistance, and hepatic FA composition in context of hepatic steatosis, and it needs to be addressed in the future experiments. 

## Figures and Tables

**Table 1:** Studies showing that *trans*-10, *cis*-12 CLA induced significant (*P* < 0.05) increases or decreases, or where there was no change (*P* > 0.05) in body, adipose, and liver weights and liver lipid concentration.

Species	Change	Body weight	Adipose tissue	Liver weight	Liver lipids
Mice^1^	Increase	—	—	24 (92)	19 (515)
	Decrease	21 (31)^ 2^	29 (666)	—	—
	No change	16	—	2	2
Rats^3^	Increase	—	—	—	1 (25)
	Decrease	—	1 (23)	—	4 (19)
	No change	11	3	8	4
Hamsters^4^	Increase	—	—	8 (20)	—
	Decrease	2 (14)	11 (20)	—	3 (37)
	No change	11	2	2	5
Humans^5^	Increase	—	—	—	—
	Decrease	2	6	—	—
	No change	11	13	—	—

^1^Studies used: [22, 25, 27–48].

^2^Number of observations (mean percent change).

^3^Studies used: [49–53].

^4^Studies used: [54–62].

^5^Studies used: [63–81].

**Table 2:** Studies showing that *trans*-10, *cis*-12 CLA induced significant (*P* < 0.05) increases (↑), decreases (↓), or no change (*↔*) (*P* > 0.05) in hepatic gene expression and circulating levels of insulin, adipokines, and TNF-*α*. Genes are classified based on their ascribed function.

	Mice^1^			Rats^2^			Hamsters^3^		
	↑	↓	*↔*	↑	↓	*↔*	↑	↓	*↔*
Lipogenesis									

* ACC*	5 (126)^4^	—	1	—	—	—	1 (99)	—	1
* FASN*	7 (243)	—	1	—	1 (50)	2	—	—	2
* SCD1*	2 (150)	—	3	—	1 (80)	—	—	—	—
* SREBP-1c*	3 (53)	—	2	—	1 (40)	4	—	—	3
* PPAR-*γ**	2 (200)	—	—	—	—	2	—	—	—
* ME*	5 (205)	—	—	—	—	—	—	—	—

FA uptake, secretion, and oxidation									

* CPT1*	4 (107)	1 (59)	1	—	—	—	—	—	2
* ACO*	5 (117)	—	1	2 (130)	—	4	—	—	2
* PPAR-*α**	—	1 (53)	—	1 (125)	—	—	—	—	3
* FAT/CD36*	3 (533)	—	—	—	—	—	—	—	—
* LPL*	—	—	1	—	—	—	—	—	1

Insulin, adipokines, and TNF*α*									

Insulin	12 (2492)	1 (29)	3	—	—	3	—	—	1
Adiponectin	—	6 (77)	5	—	—	—	—	—	—
Leptin	—	10 (71)	—	—	—	1	—	—	—
TNF-*α*	—	4 (32)	1		1 (44)	2	—	—	—

↑, ↓, *↔*; increase, decrease or no changes respectively.

^1^Studies used: [27, 29–35, 45, 95, 96].

^2^Studies used: [49–52, 97].

^3^Studies used: [54, 56, 59, 61].

^4^Number of observations (mean percent change).

*ACC: acetyl CoA carboxylase, FASN: fatty acid synthase, SCD1: stearoyl CoA desaturase-1, SREBP-1c: sterol regulatory element-binding protein-1c, PPAR-*γ**: peroxisome proliferator activated receptor-**γ**, *ME: malic enzyme, CPT1: carnitine palmitoyl transferase 1, ACO: acyl-CoA oxidase, PPARα: peroxisomal proliferator activated receptorα; FAT/CD36: fatty acid translocase, LPL: lipoprotein lipase*.

**Table 3:** Summary of literature studies on amelioration of CLA induced hepatic steatosis.

			% Added dietary CLA				
Reference	No. per treatment	Study days	CLA Mix	*trans*-10 *cis*-12	Treatment	Treatment dose, %^1^	Observations
[136]	3 to 6	28	2.0	0.95	Leptin	5 *μ*g/d	↓ Hepatic steatosis, ↑ insulin sensitivity,
[40]	5 to 14	30	1.0	0.72	Leptin	5 *μ*g/d	↑ insulin sensitivity, ameliorated hepatic steatosis
[49]	5	28	1.5	0.60	Rosiglitazone	10 mg/kg BW	↑ Insulin sensitivity, prevented depletion of epididymal adipose tissue
[35]	10	42	2.0	1.00	Rosiglitazone	10 mg/kg BW	↓ Hepatic TG content, ↓ hepatic lipogenesis,↑ serum leptin and adiponectin, prevents lipodystrophy
[140]	7	28	3.0	0.98	Arachidonic acid	1, 2	↓ Induction of hepatic steatosis, ↑ liver PGE2, ↑ epididymal adipose
[44]	7	28	—	1.20	*γ*-Linolenic acid	5	↓ Hepatic steatosis, ↑ PGE_2_
[38]	10	56	—	0.50	Flax seed oil (*α*- Linolenic acid)	0.39	↓ Steatosis, ↑ n-3 and n-6 PUFA in liver
[27]	7 to 8	22	1.0	0.50	Fish oil	1.5, 3, 6	↑ Leptin and Adiponectin, ↓ Insulin, ↓ TG in liver, ↑ fat pad
[141]	10	105	1.0	0.50	Pine oil	7.5	Serum insulin levels stabilized over 3 weeks
[135]	5 to 6	100	1.0	0.35	34% dietary fat		Normal plasma insulin levels, ↑ liver weight
[45]	6	28	2.0	0.74	DHA	0.5	↓ Fatty liver, ↓ FA synthesis, plasma leptin, and adiponectin unaffected
[142]	10	56	—	0.50	DHA, EPA	0.5, 0.5	Prevented hepatic steatosis, partially restored plasma leptin, only DHA restored plasma adiponectin

^1^Percentage in the diet except wherever noted.
